# Characterization of a New Protein Family Associated With the Silica Deposition Vesicle Membrane Enables Genetic Manipulation of Diatom Silica

**DOI:** 10.1038/s41598-017-13613-8

**Published:** 2017-10-18

**Authors:** Benoit Tesson, Sarah J. L. Lerch, Mark Hildebrand

**Affiliations:** Marine Biology Research Division, Scripps Institution of Oceanography, University of California San Diego, La Jolla, California, United States of America

## Abstract

Diatoms are known for their intricate, silicified cell walls (frustules). Silica polymerization occurs in a compartment called the silica deposition vesicle (SDV) and it was proposed that the cytoskeleton influences silica patterning through the SDV membrane (silicalemma) via interactions with transmembrane proteins. In this work we identify a family of proteins associated with the silicalemma, named SAPs for Silicalemma Associated Proteins. The *T*. *pseudonana* SAPs (TpSAPs) are characterized by their motif organization; each contains a transmembrane domain, serine rich region and a conserved cytoplasmic domain. Fluorescent tagging demonstrated that two of the TpSAPs were localized to the silicalemma and that the intralumenal region of TpSAP3 remained embedded in the silica while the cytoplasmic region was cleaved. Knockdown lines of TpSAP1 and 3 displayed malformed valves; which confirmed their roles in frustule morphogenesis. This study provides the first demonstration of altering silica structure through manipulation of a single gene.

## Introduction

Diatoms are well known for their ability to synthesize an exoskeleton through the polymerization of silicic acid into silica. Diatom cell walls, also called frustules, consist of two halves, called theca, which are composed of valves (the “top” and “bottom”) connected to a series of overlapping girdle bands which typically encircle each valve and connect the theca. These cell wall components have a diverse variety of shapes and structures which serve putative roles in protection, light modification and buoyancy^1^. Additionally these structures are applicable to nanotechnology in fields such as drug delivery^2^, biosensing^3^, and solar cells and batteries^4^.

The centric diatom *Thalassiosira pseudonana* has been developed as a model species to study the formation of silica cell walls. The distinct stages of valve formation in *T*. *pseudonana* have been described in detail^5^. Initially, a base-layer is formed which defines the x/y dimensions of the valve. The base layer consists of silica ribs radiating from a point called the pattern center, usually located near the center of the valve, and fields of pores between the ribs. Formation of particular substructures, such as portulae, is initiated during base layer formation. After base layer formation, silicification proceeds in the z-axis direction, with deposition of silica particles on top of and in short segments between the ribs, forming an interconnected network characteristic of the distal surface of the valve.

In the last decade great progress has been made in understanding the conserved molecular mechanisms involved in silicic acid polymerization^6–8^. Silica structure formation takes place inside a membrane-bound compartment, the Silica Deposition Vesicle (SDV)^9^. Proteins (silaffins, silacidins and cingulins) and long chain polyamines (LCPAs) are involved in silicic acid polymerization^7,10–12^. Silaffins and silacidins are soluble proteins targeted to the SDV lumen where they are thought to self-assemble with LCPAs and catalyze silica polymerization. Recently, insoluble organic matrices comprised of proteins and polysaccharides have been isolated from diatom cell walls^11,13^. These matrices precisely mimic the detailed structures and patterns of the proximal cell wall surface, suggesting that they are involved in shaping the silica^13^. Multiple silica polymerizing proteins have been found embedded within the insoluble organic matrix associated with *T*. *pseudonana* cell walls^10^.

The cytoskeleton is also involved in the control of cell wall morphogenesis. Microtubule networks are tightly associated with the SDV and may be involved in shaping and strengthening it, as well as determining the locations of cell wall features^14–16^. Indeed, inhibition of microtubule formation alters overall valve formation and patterning of microscale structures^17,18^. A peripheral ring of actin microfilaments appears to define the extent of SDV growth. Additionally during cell wall morphogenesis actin filaments were found interdigitated with forming silica structures or matching the detailed mesoscale patterns of the valves^14^. These cytoskeletal elements, located in the cytoplasm, are thought to impact the patterning of silica inside the SDV lumen, a process that would require the transmission of organizational patterns across the SDV membrane (the silicalemma)^14,17^. A model for this process implicates putative silicalemma spanning proteins which simultaneously interact with cytoskeletal elements in the cytoplasm and silica polymerizing determinants in the SDV lumen, resulting in a replication of cytoskeletal patterns in silica structures^19^. These putative proteins have not yet been identified.

A previous study monitored changes in transcript expression patterns using whole genome microarrays on a synchronized culture of *T*. *pseudonana* to identify a host of genes proposed to be involved in silica cell wall formation. This study identified 485 genes with similar expression patterns to Silaffin 3, a known silica polymerization protein; these genes were named the Silaffin Like Response Genes (SLRGs)^20^. A subset of 13 unknown genes within the SLRG dataset contained a predicted ER signal peptide and transmembrane (TM) domain, identifying them as candidates to transmit organizational patterns from the cytoplasm into the SDV lumen^20^. In this report, based on investigations of a single protein from the SLRG subset, we identify and characterize three proteins with similar features in *T*. *pseudonana*¸ which constitute a new family of proteins: the Silicalemma Associated Proteins (SAPs). The SAPs are conserved in multiple diatom species and are defined by their distinctive sequence organization and C-terminal conserved domain. The common motif organization and alterations of silica structure in TpSAP1 and 3 knockdown lines are consistent with the SAP family playing a role in frustule morphogenesis.

## Material and Methods

### Culture conditions

*Thalassiosira pseudonana* (Hustedt) Hasle et Heimdal (CCMP1335), was grown in ASW^21^ medium under continuous light at 19 °C while bubbled with air. For imaging, the culture was synchronized in order to enrich for cells making valves as described previously^22^.

### Preparation of cleaned frustules

Two methods were used in frustule preparation for SEM. One method harvested cells by centrifugation, suspended in 1% SDS, 0.1 M EDTA and heated at 50 °C, this process was repeated three times. Then pellets were washed in MilliQ water, acetone and again three times in MilliQ water. In the second method a modified version of an acid cleaning method^23^ was implemented with the modification that all washes were done with MilliQ water and the full method was repeated twice before final suspension in ethanol.

### Sequence comparison and analyses

BLASTp searches were done querying Thaps3_25736 (TpTpSAP1) against the NCBI Reference Sequence (RefSeq) v51, the *Fragilariopsis cylindrus* genome^24^, the *Cyclotella cryptica* genome^25^ and the Moore Foundation Marine Microbial Eukaryote Transcriptome Sequencing Project dataset^26^, in order to identify homologous sequences. Queries for *F*. *cylindrus* were done on filtered models with an expected threshold of ten; all other queries were done with pre-set search parameters. Sequence alignments and trees were generated using CLC Main Workbench 6.7.1, Geneious 6.1^27^ and ClustalW^28^. The theoretical mass, isoelectric point, and amino acid composition of the TpSAPs were computed using ProtParam from the EXPASY online server^29^. Signal peptide and transmembrane domains were predicted using SignalP^30^ and TMHMM^31^ respectively. The potential sites of post-translational modification were predicted using DictyOGlyc 1.1^32^, NetCGlyc 1.0^33^, NetNGlyc 1.0^34^, YinOYang 1.2^35,36^, and NetPhos 2.0^37^ from the EXPASY online server.

### RNA sequencing

RNAseq sequencing data was generated on a silicon-starvation synchronized culture of *T*. *pseudonana*. Axenic cultures of *Thalassiosira pseudonana* (CCMP1335) were synchronized as previously described^20,22^. Prior to and then every hour after silicate addition, 750 ml of culture was removed, treated with cycloheximide (20 μg ml^−1^), and harvested. Total RNA from biological duplicate samples was isolated using RNAzol^38^. RNAseq libraries were prepared using the Illumina TruSeq mRNA Sample Prep kit (Illumina). RNAseq library preparation and sequencing was performed by courtesy of Dr. Matteo Pellegrini at University of California Los Angles (UCLA) using procedures as detailed by Traller *et al*.^25^. Briefly, RNAseq libraries were constructed using the Illumina TruSeq mRNA Sample Prep kit (Illumina). Sequencing was then performed on a HiSeq. 2000 sequencer (Illumina) using a mixture of 50 + 50 nt paired end reads and 100 nt single end reads. The raw sequence data was processed as described in Traller *et al*.^25^.

### Diatom constructs and transformation

Localization constructs were generated using either the fcp promoter or native promoters to control expression. For expression under the fcp promoter, a *T*. *pseudonana* Gateway™ destination vector (pMHL_79) was created by inserting a reading frame B cassette between the fcp promoter and eGFP in pTpFcpGFP (Fig. S1)^39^. Genes of interest were PCR amplified (Table S1) and cloned into pMHL_79. An additional construct under fcp promoter/terminator control was created with Thaps3_25807 (TpTpSAP3), by inserting eGFP between the signal peptide and the serine rich region (Fig. S2).

For expression under the control of native promoters, DNA fragments, including promoter and full-length coding sequences devoid of stop codons, were amplified by PCR from *T*. *pseudonana* genomic DNA and cloned into the destination vector pMHL_71 with eGFP at the end of the coding sequence to create transformation vectors. We included 1,000 bp upstream of the first methionine and 500 bp downstream of the stop codon to encompass the promoter and terminator.

RNAi and antisense knockdown constructs, where the knockdown sequence and selectable marker expression were controlled by fcp, were generated using Gateway™ cloning technology as previously described^40^. *T*. *pseudonana* RNAi and antisense sequences were isolated via PCR using the primers shown in Table S1. Antisense regions spanned 560 and 448 base pairs in length for Thaps3_25736 (TpTpSAP1) and Thaps3_25807 (TpTpSAP3) respectively.

*T*. *pseudonana* was transformed with the resulting vectors using particle bombardment^41,42^. Localization constructs were co-transformed with pTpFcpNAT^39^. Resistant colonies were selected from NEPC or ASW agar plates containing 100 µg/ml nourseothricin. Knockdown clones were screened using PCR to confirm construct integration.

### Fluorescent staining of forming silica structures

Silica incorporation was visualized by the addition of 100 ng mL^−1^ PDMPO ([2-(4-pyridyl)-5-((4-(2-dimethylaminoethylamino- carbamoyl)methoxy)phenyl)oxazole] to the culture medium^17,43^.

### Fluorescence microscopy

Cells were imaged using a Zeiss AxioObserver inverted microscope equipped with an Apotome. The filter sets used were Zeiss #21HE (Ex 387/15 nm, FT 409, Em 510/90 nm) for PDMPO, Zeiss #38HE (Ex 470/40 nm, FT 495, Em 525/50 nm) for GFP and Zeiss#05 (Ex 395–440 nm, FT 460 nm, Em 470 nm LP) for chlorophyll. Images were acquired with 63x/1.4 objective oil immersion plan APO and processed using Axiovision 4.7.2.

Measurements of GFP relative fluorescence intensity in the intracellular compartments were performed using the Axiovision software (Zeiss) on images acquired with the same exposure time. Images were acquired of cells harvested from an exponentially growing culture and after 4hrs silicon starvation (n = 24).

### Scanning electron microscopy and micrograph analysis

Cleaned samples were coated with gold/ palladium and imaged using a Philips XL 30 ESEM (UCSD, Calit2 Nano3 facilities). Micrograph analysis was done on valves laying on level surfaces. Relative variability in the distal surface roughness of valves was quantified by averaging the standard deviation of grayscale intensity from two peripheral regions of the valve surface using ImageJ (n = 20)^44^. Grayscale intensity in SEM micrographs is indicative of sample topology, therefore greater variability in grayscale intensity indicates a relatively rougher surface texture. One way analysis of variance (ANOVA) with a correction for unequal variance was performed on relative roughness data followed by a Games-Howell post hoc test.

### Protein extraction and Western blot

Proteins were extracted by boiling for 10 min in sample buffer (Biorad) and centrifuging at 10,000 g. Protein quantity was measured using the DC protein assay kit (Biorad). Equivalent amounts of protein were loaded on each lane of a Mini-protean TGX precast gel (Biorad). Proteins were transferred to a nitrocellulose membrane using a semi dry transfer system (Biorad transBlot turbo). Rabbit eGFP primary antibody and HRP-conjugated goat anti rabbit secondary antibody were detected using the SuperSignal West pico chemiluminescent substrate (Thermo Fisher). Densitometry analysis of Western blot bands was completed using FIJI^45^.

## Results

### Identification and characterization of the protein family

**Figure 1:**
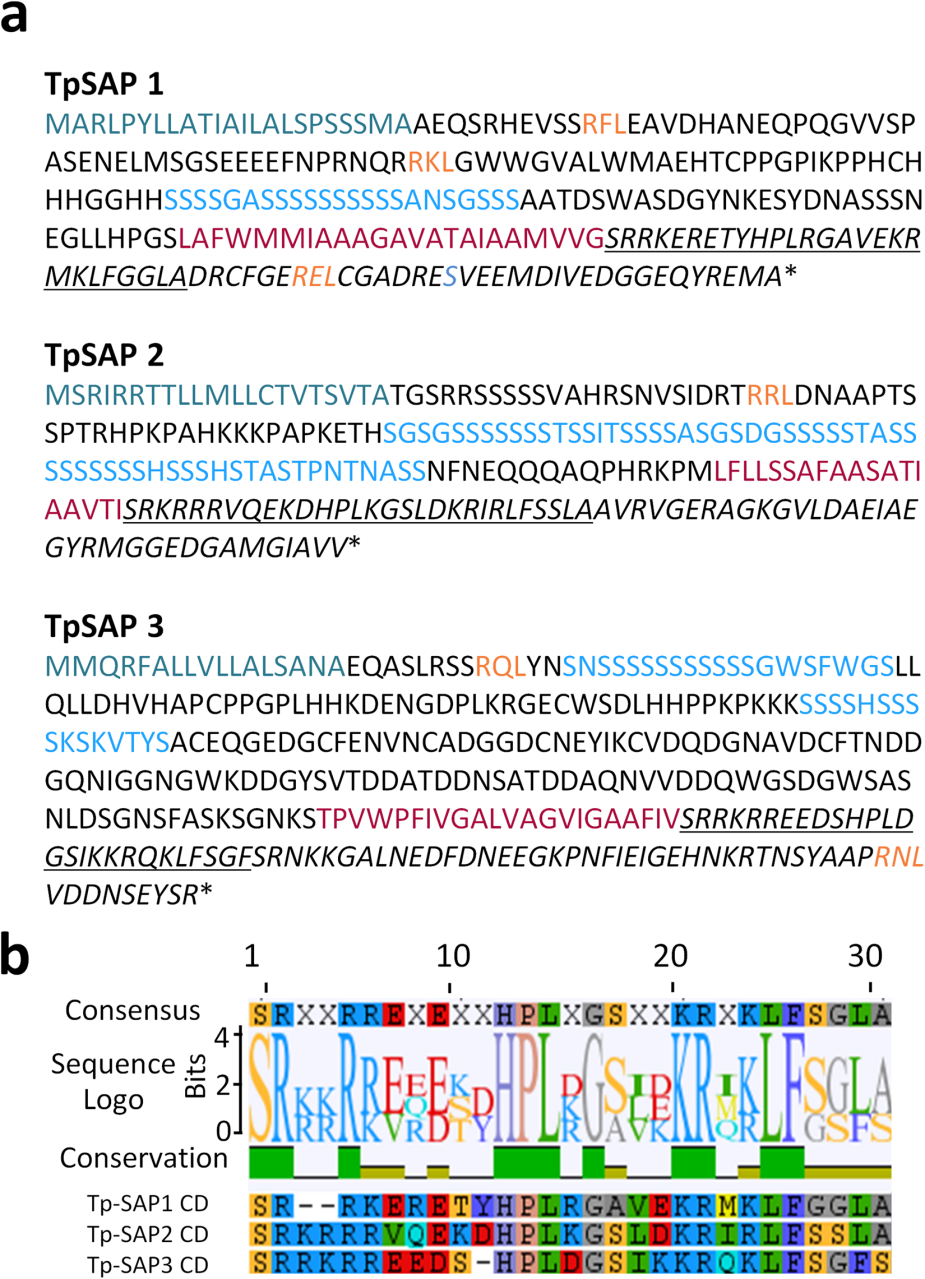
TpSAP protein sequences and alignment. (**a**) TpSAP protein sequences. Signal peptides are in dark teal, RXL domains in orange, serine rich regions in blue, transmembrane domains in red, conserved cytoplasmic domains are underlined and italicized regions are predicted to be cytoplasmic. (**b**) Alignment of TpSAP conserved cytoplasmic domains.

Within the previously identified set of Silaffin Like Response Genes (SLRGs)^20^ we identified a gene encoding a protein (Thaps3_25736) with predicted features consistent with silicalemma association, which we named TpSAP1 (Silicalemma Associated Protein 1). In addition to having a silaffin-like expression pattern, TpSAP1 had a predicted ER signal peptide and a single transmembrane domain (Fig. 1). BLAST searches identified two other similar proteins in the *T*. *pseudonana* genome, two proteins each in the *F*. *cylindrus* and *T*. *oceanica* genomes and three in the *C*. *cryptica* genome (Table S2)^24,25,46,47^. These proteins displayed similar overall motif arrangements and some sequence similarity, including a conserved domain adjacent (C-terminal) to the transmembrane domain (Fig. S3). In addition, matches were observed to sequences in eight other centric species in the Marine Microbial Eukaryote Transcriptome Sequencing Project (MMETSP) dataset (Fig. S4a)^26^. The MMETSP dataset hits demonstrated the greatest sequence conservation in and around the conserved domain (Fig. S4b).

The overall sequence similarity between TpSAPs was low and mostly restricted to specific regions (Fig. 1 and S3). Despite a low degree of sequence similarity, these proteins had a characteristic organization which made them easily identifiable. They possessed a signal peptide, and a single transmembrane domain which separated two portions of the proteins; the longer N-terminal portion was predicted to be localized inside the SDV (assuming silicalemma localization, see below) and the shorter C-terminal sequence was predicted to be exposed to the cytoplasm (Fig. 1a). The C-terminal section contained a 28 amino acid segment with a conserved sequence localized just after the predicted transmembrane domain (Figs 1, S3, S4b). The N-terminal section contained a segment highly enriched in serine residues, of variable length, located between the signal peptide and the transmembrane domain (Figs 1 and S3). TpSAP1 had a 23 amino acid long region containing 18 serines and was comprised of 15.5% serine overall, TpSAP2 had a 51 amino acid long region containing 36 serines (24.8% overall), and TpSAP3 had 20 and 16 amino acid long regions containing 14 and 10 serines respectively (15% overall). TpSAP1 and 3 had acidic isoelectric points while TpSAP2 was basic (Table S2). Additionally, we identified RXL domains, a proteolytic cleavage site previously identified in biosilica associated proteins^7,11,12^, in the N-terminal portion after the signal peptide in all TpSAPs and in the C-terminal portion after the conserved domain in TpSAP1 and 3 (Fig. 1).

### Expression and localization of the TpSAPs

**Figure 2:**
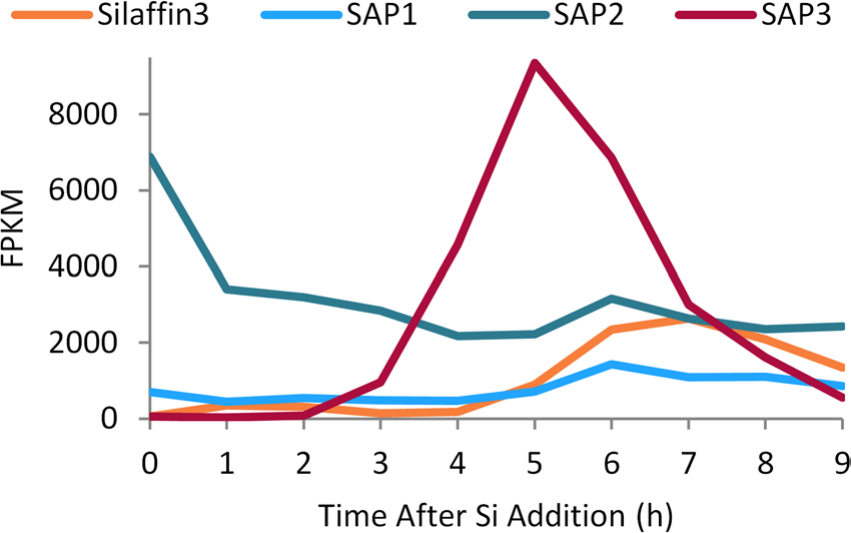
RNAseq-based expression profiles of TpSAPs and silaffin 3 during synchronized culture progression.

We examined transcript changes for the three TpSAPs using RNAseq data which provided better temporal resolution than our previous microarray data^20^. In this experiment, Silaffin 3 transcript levels, diagnostic of the period of valve formation^23^ were induced at 6 and 7 h and then decreased (Fig. 2). The expression profile of TpSAP1 was similar to Silaffin 3, increasing at 6 h when valve synthesis occurred and then slowly decreasing. TpSAP3 transcripts increased earlier and reached a maximum at 5 h, showing the largest magnitude change and highest transcript level of all the TpSAPs as well as being substantially greater than Silaffin 3. The expression profile of TpSAP2 differed from the other TpSAPs, exhibiting maximal expression at 0 h then decreasing and remaining constant with the exception of a small peak at 6 h (Fig. 2).

We generated C-terminal GFP fusion proteins to determine the localization of the TpSAPs, initially using a strong, constitutive promoter (fucoxanthin chlorophyll a*/c* binding protein, fcp) to aid in visualization, and then using native promoter/terminator cassettes to verify that over expression did not induce artifacts.

**Figure 3:**
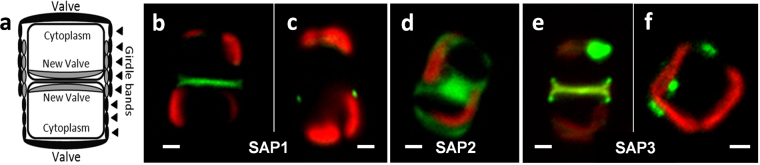
Intracellular localization of TpSAP C-terminal GFP fusion proteins. (**a**) Schematic representation of a dividing diatom. (**b** and **c**) TpSAP1-GFP fusion protein under fcp promoter control localized to the site of (**b**) valve and (**c**) girdle band formation. (**d**) TpSAP2-GFP under native promoter control localized to the cytosol. (**e** and **f**) TpSAP3-GFP under native promoter control localized to the site of (**e**) valve and (**f**) girdle band formation. (GFP is green, chlorophyll autofluoresence is red). Scale bars are 1 µm.

The TpSAP1–GFP fusion protein was localized to the sites of valve and girdle band formation but no fluorescence was observed in the mature frustules (Fig. 3b,c). No other intracellular localization was observed. Using a native promoter and terminator we observed similar localization, however GFP fluorescence was very dim and difficult to image (not shown).

Under the native promoter, theTpSAP2-GFP fusion protein was observed in the cytosol (Fig. 3d).

**Figure 4:**
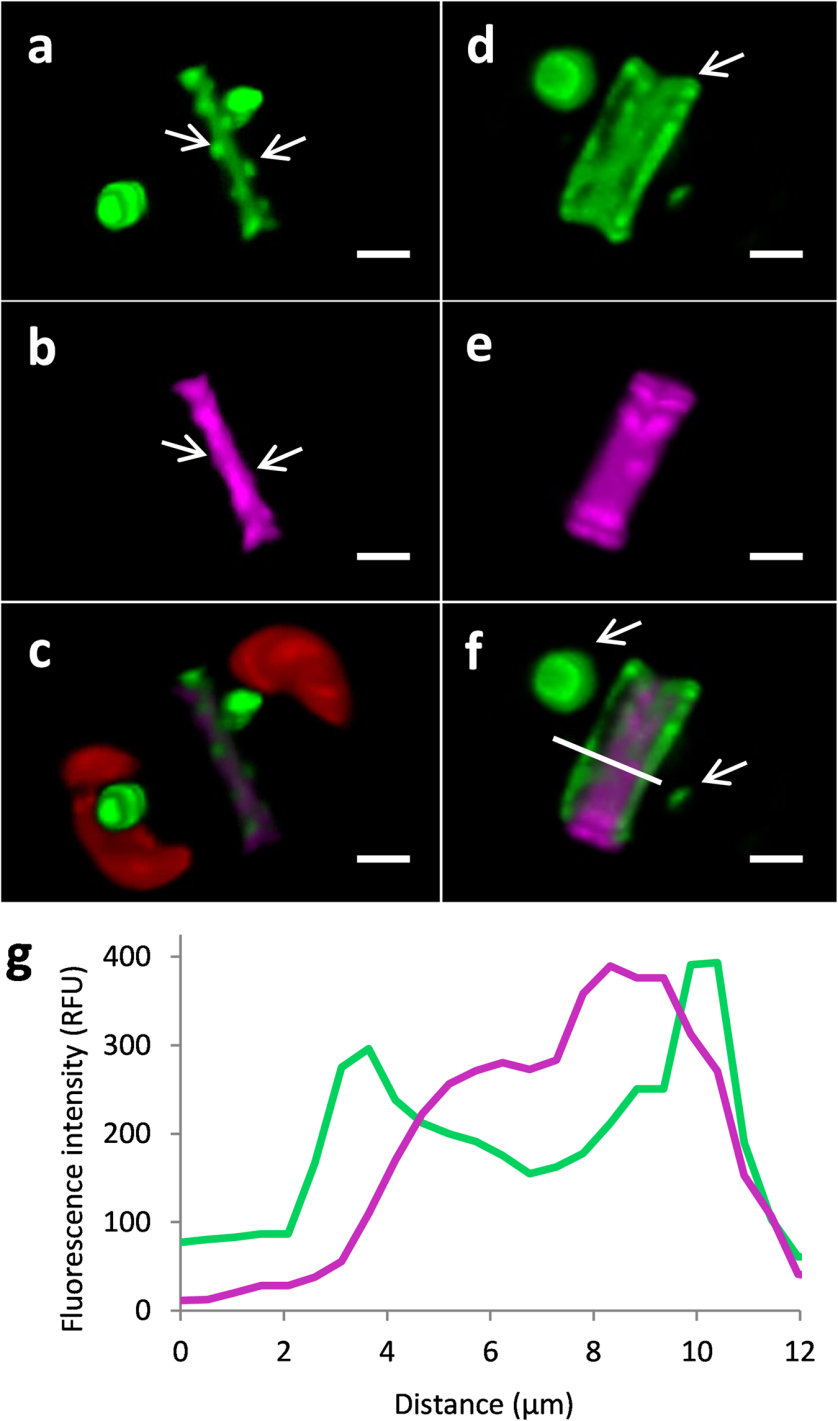
3D reconstruction of TpSAP3-GFP C-terminal fusion protein associated with forming valves generated from z-stack images. Fusion protein expressed under native promoter control. Each column displays a separate cell in an early (**a**,**b**,**c**) and later (**d**,**e**,**f**) stage of valve formation. (PDMPO staining of silica is pink, GFP is green, and chlorophyll autofluoresence is red). (**a** and **d**) GFP, (**b** and **e**) silica, (**c** and **f**) merged, (**g**) profile of fluorescence intensity corresponding to a cross section done at the white line in (**f**). Arrows point to portulae in (**a** and **b**), to the rim of the valve in (**d**) and to the fluorescent compartments in (**f**). Scale bars are 1 µm.

**Figure 5:**
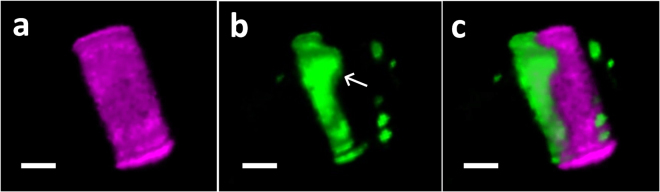
3D reconstruction of TpSAP3-GFP C-terminal fusion protein associated with forming girdle bands generated from z-stack images. Adjacent girdle bands are shown, one mature and one forming. PDMPO staining of silica is pink and GFP is green. (**a**) Both girdle bands, PDMPO stained. (**b**) GFP localization with forming girdle band. Arrow indicates location of ligula. (**c**) Merged image. Scale bars are 1 µm.

Similarly toTpSAP1, TpSAP3-GFP was localized to sites of forming valves and girdle bands under native promoter-controlled expression (Figs 3e,f, 4). In addition, roughly spherical compartments exhibited GFP fluorescence (Figs 3e,f, 4). More rigorous localization of TpSAP3-GFP was performed by staining silica with PDMPO (Figs 4 and 5). TpSAP3 generally co-localized with forming silica (Figs 4, 5). In particular, in the valves one can distinguish bright dots corresponding to the location of portulae (Fig. 4a–c). At a later stage in valve formation, GFP fluorescence was enriched at the outer rim of the forming valves (Fig. 4d–f). A fluorescence intensity cross-section showed that TpSAP3 was present on the outside edge of the forming silica (Fig. 4g). We also observed GFP fluorescence associated with the girdle bands during their formation (Fig. 5). The images in Fig. 5 show GFP fluorescence localized to an entire forming girdle band, even defining the ligula. Based on the lack of GFP fluorescence in mature silica structures, the second girdle band only stained with PDMPO is interpreted to be a mature structure where GFP has been removed (Fig. 5).

Fluorescent spherical compartments were also observed in the cell’s cytoplasm (Figs 3, 4). During girdle band formation, we observed a single fluorescent intracytoplasmic compartment (Fig. 3f). Observation of a number of cells revealed that when cells were dividing and making new valves a fluorescent compartment was localized to each daughter cell (Fig. 4a,c,d,f, S5). These compartments were not always visible depending on the optical plane presented (Figs 3e, 5). The origin and precise localization of this compartment is not known, however DAPI staining suggested that this compartment was close to the nucleus in cells that were dividing (Fig. S5). Fluorescence intensity in this intracellular compartment increased during silicon starvation, suggesting an accumulation of protein. After 4 hours of silicon starvation, relative fluorescence intensity in this compartment was significantly higher than in an exponentially growing culture (Fig. S6).

**Figure 6:**
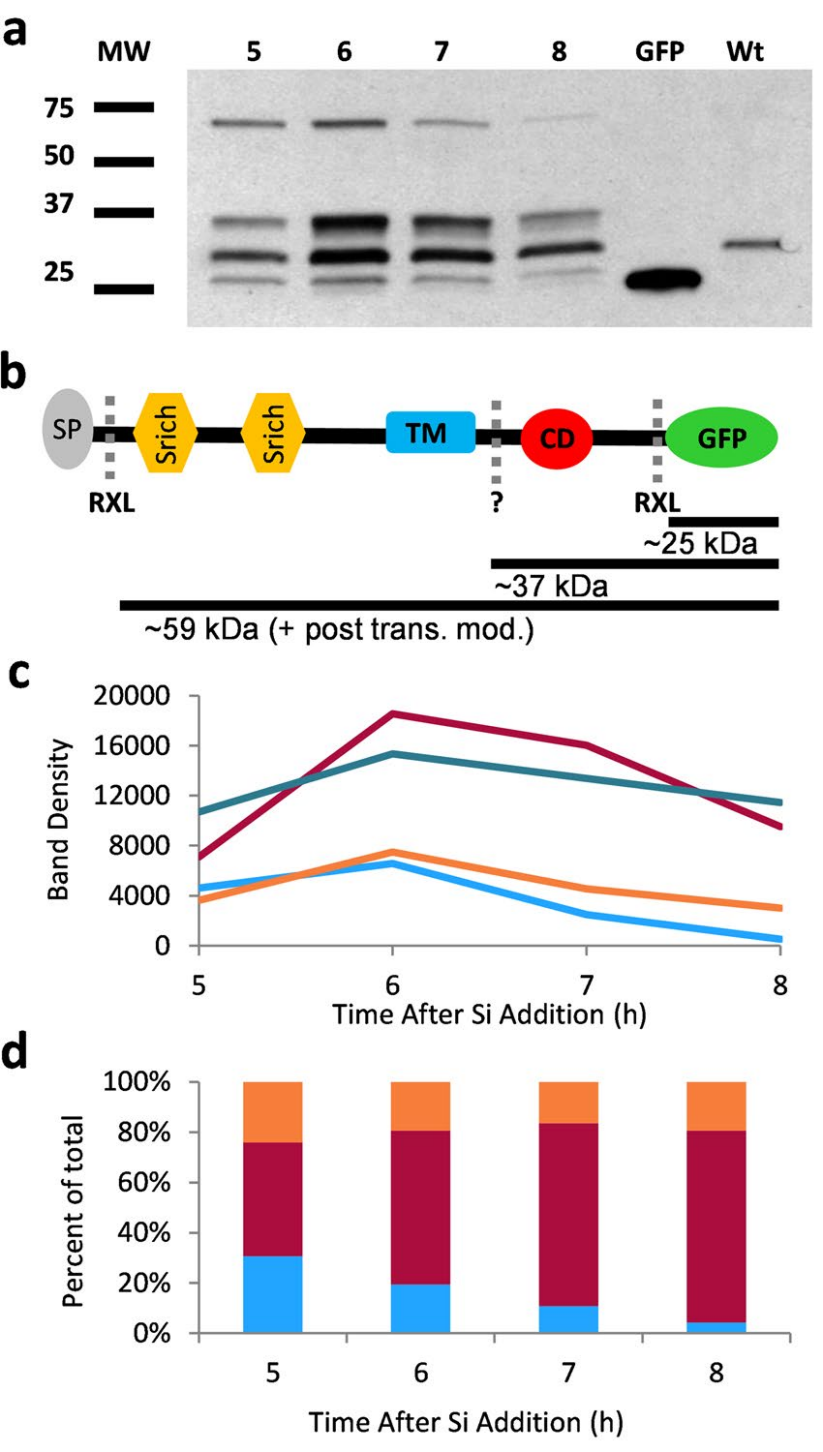
Western blot and cleavage fragment analysis of cells expressing TpSAP3-GFP C-terminal fusion protein. (**a**) Western blot (WB) with anti GFP antibody on whole cell protein extracts from a synchronized culture expressing the TpSAP3-GFP fusion protein under native control at hours 5–8. (**b**) Schematic representation of TpSAP3-GFP protein with resulting peptide sizes after cleavage (SP: Signal Peptide, Srich: Serine rich region, TM: transmembrane domain, CD: Conserved Domain, RXL: RXL domain sites, dashed lines indicate known or putative (?) cleavage sites). (**c**) Quantification by densitometry of WB bands over time. (**d**) Quantification by densitometry of the amount of cleavage product relative to the total at each time point. 70 kDa band (blue), 37 kDa band (red), 30 kDa (native peroxidase) band (dark teal) and 25 kDa band (orange).

The lack of TpSAP1 and 3-GFP fluorescence associated with the mature frustule suggested the possibility of proteolytic cleavage, removing GFP from the C-terminal portion of the protein. In order to investigate this possibility we performed a Western Blot with a GFP antibody on a synchronized culture of a clone expressing native TpSAP3-GFP (Fig. 6a). We observed bands of different molecular weights at approximately 25, 37 and 70 kDa. Another band at 30 kDa was also found in wild type and was determined to be a native peroxidase which maintains activity in the gel (unpublished). The theoretical molecular weight of the TpSAP3-GFP fusion protein was 58.9 kDa after removal of the pro-peptide (Fig. 6b). This was less than the highest molecular weight band at approximately 70 kDa, which may suggest that the protein was post-translationally modified, a common trait of silica-associated proteins^7^. Using posttranslational prediction tools, 51 potential phosphorylation sites were found for TpSAP3 (Fig. S7, Table S2). Additionally, putative glycosylation sites were also found (Fig. S7, Table S2). Densitometry analysis revealed that the total amount of cleavage product was maximal at 6 h and that the 37 kDa cleavage product was the most abundant (Fig. 6c). The amount of peroxidase band also changed over time but with different magnitudes when compared to changes in the TpSAP3-GFP cleavage fragments (Fig. 6c). Quantification of each band relative to the total cleavage product at each time point showed that the 70 kDa and 37 kDa products changed inversely over time, consistent with a precursor/product relationship. The 25 kDa doublet was analyzed as a single band and accounted for a consistent percentage of the total over time (Fig. 6d).

**Figure 7:**
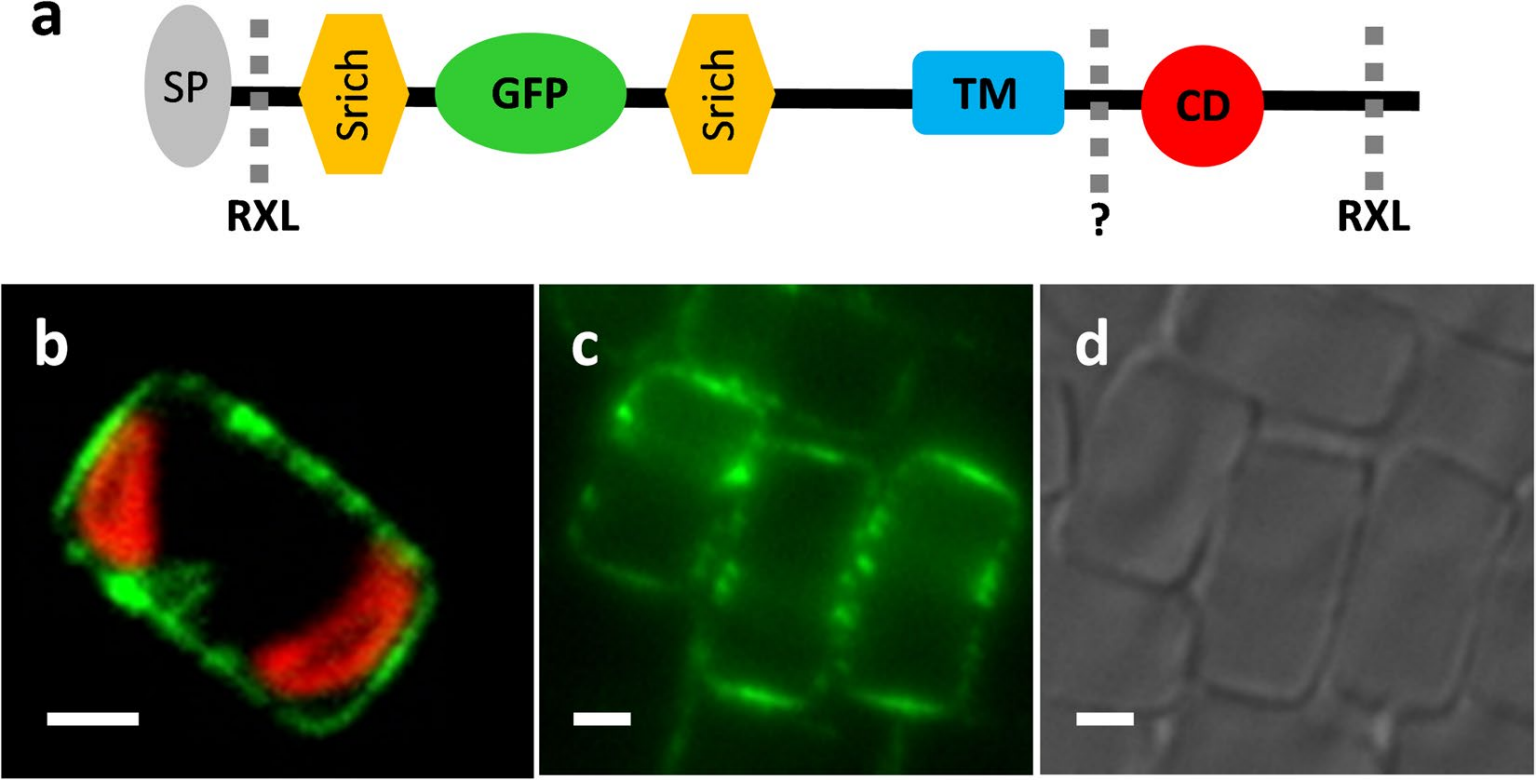
Diagram and localization of TpSAP3-GFP fusion protein when GFP is inserted in the intralumenal region of the protein within the serine rich region. (**a**) Schematic representation of internal GFP fusion protein (SP: Signal Peptide, Srich: Serine rich region, TM: transmembrane domain, CD: Conserved Domain, RXL: RXL domain sites, dashed lines indicate known or putative (?) cleavage sites) (**b**) Intact cell expressing fusion protein. (**c**) SDS cleaned frustules GFP fluorescence and (**d**) corresponding differential interference contrast image. (GFP is green and chlorophyll autofluorescence is red). Scale bars are 2 µm.

The presence of low molecular weight bands and putative cleavage sites near the C-terminus of the protein confirmed that GFP was removed. To verify whether a portion of the protein remained associated with the mature cell wall we inserted GFP into the N-terminal portion of TpSAP3 between the pro-peptide and the transmembrane domain, 86 amino acids after the first methionine (Fig. 7, S2). With this construct, GFP fluorescence was observed associated with the mature cell wall and remained associated after SDS cleaning (Fig. 7)^48^. This is consistent with localization of the N-terminal portion inside the SDV lumen and a tight association with the silica. In contrast to the C-terminus TpSAP3-GFP fusion, no fluorescence associated with intracellular vesicles was observed.

### Effect of TpSAP1 and 3 knockdowns

To further investigate the role of TpSAPs during frustule morphogenesis, we generated TpSAP1 and TpSAP3 (the two TpSAP proteins associated with forming silica structures) knock down lines, using RNAi and RNA antisense approaches. To evaluate the effects of target knockdown a minimum of 20 valves were observed using SEM for four TpSAP1 and six TpSAP3 transgenic lines.

This screening approach was used due to the limitations of Western Blots and RT-qPCR in the context of evaluating knockdown. The strong likelihood of extensive post translational modifications to these target proteins indicates that specific antibodies used for quantitation could not be made. Previous work also demonstrates a lack of correlation between transcript levels monitored by RT-qPCR and the extent of knockdown, indeed more abundant transcripts have been found in knockdown lines with decreased protein content^40^, presumably due to a greater effect of knockdown on translation than transcription. We have previously established that doing a phenotypic screen on a sufficient number of transgenic lines is a valid method for establishing knockdowns^49^. This approach is similar to a classical genetic screen, where a consistent phenotype in independent lines provides evidence for a genetic change.

**Figure 8:**
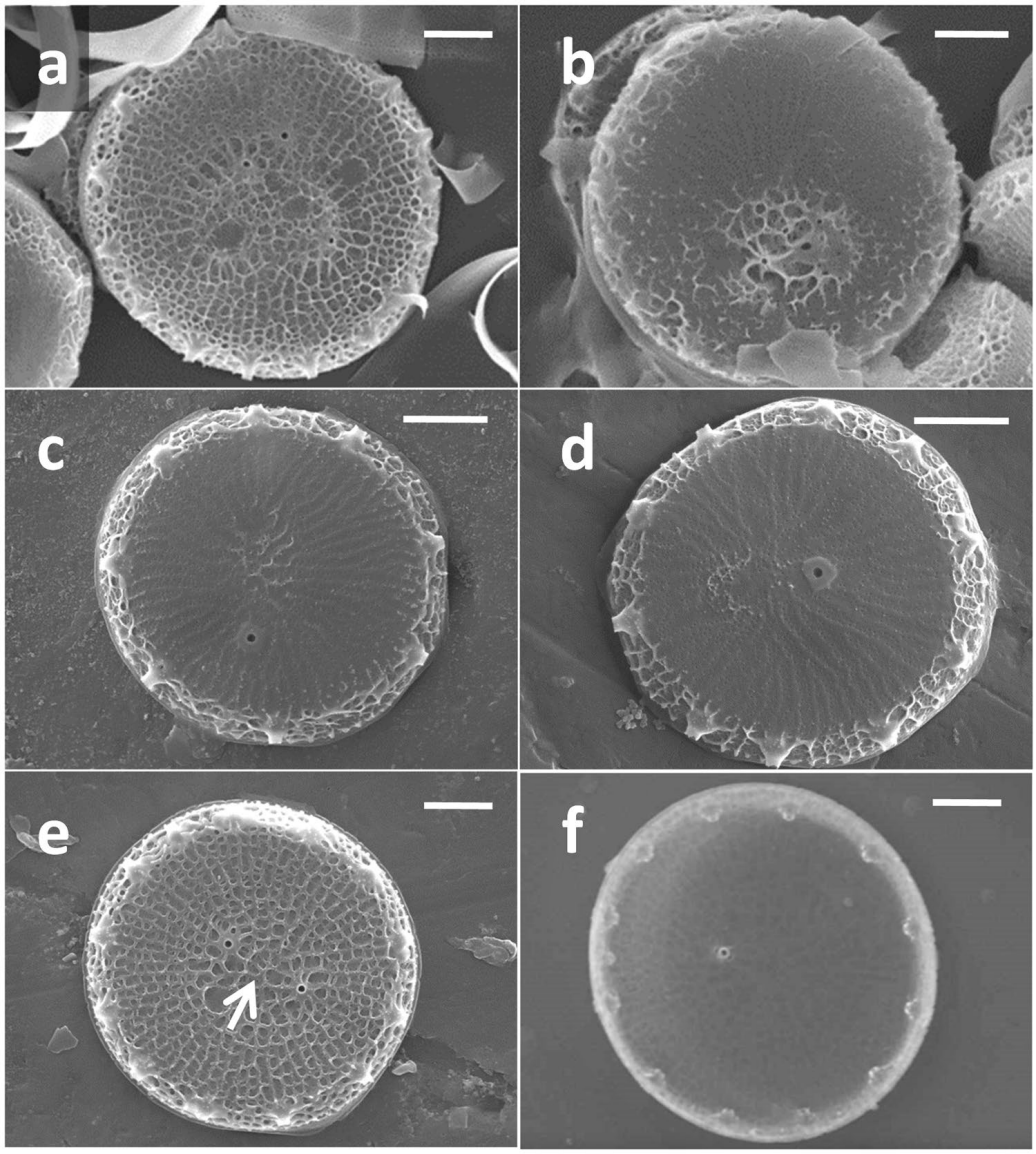
Valves displaying TpSAP1 and 3 knockdown phenotypes. (**a** and **b**) Micrographs showing distal surfaces of valves synthesized by TpSAP1 knockdown lines. (**c** and **d**) Micrographs showing distal surfaces of valves synthesized by TpSAP3 knock down lines. Micrographs of valves synthesized by wild type cells showing distal (**e**) and proximal (**f**) surface views. Arrow indicates typical location of pattern center. Scale bars are 1 µm.

Scanning electron micrographs of cleaned valves from selected knock down lines displayed consistent alterations in the distal surface silica structure (Figs 8, S8, S9). Valves from the TpSAP1 knock down lines were characterized by mislocated pattern centers as well as abnormal silicification and patterning on the distal layers (Figs 8a,b, S8). TpSAP3 knock down lines had typically-localized pattern centers, but consistently presented a reduced amount of silicification of the distal surface, leaving a nearly bare base layer in some cases (Figs 8c,d, S9). All knockdown lines except RNAi-15 displayed significantly less distal surface silicification, as evidenced by variability in distal surface texture height relative to wild type as measured by standard deviations in SEM micrograph grayscale intensity (Fig. S10). The proximal valve surface in TpSAP3 knockdowns was unaltered (Fig. S9d,p).

## Discussion

We have characterized genes encoding three proteins in *T*. *pseudonana* that share similarities in sequence and motif organization; including a predicted intralumenal N-terminal region and cytoplasmic C-terminal region separated by a transmembrane domain. Two of these proteins (TpSAP1 and 3) were found associated with forming silica structures, and additional characterization is consistent with their localization to the silicalemma. The relatively high levels of TpSAP3 transcript during valve formation (Fig. 2) are consistent with it playing a significant role in cell wall formation, this led us to characterize TpSAP3 in the most detail. By inserting GFP into the predicted intralumenal portion of TpSAP3 we showed that it remained associated with the frustule after exocytosis, even after SDS treatment (Fig. 7), indicating that the intralumenal portion of the protein is trapped within the silica matrix. These results, combined with the presence of a single TM domain and localization with forming silica structures, indicate an association of these proteins with the silicalemma. From this, we named the family silicalemma associated proteins or SAPs. Another silicalemma associated protein in *T*. *pseudonana*, Sin1, was recently discovered. Although the Sin1 and SAP sequences are distinct they share common features including a single TM domain, ER targeting, and a N-terminus embedded within the silica matrix^50^. Interestingly, Sin1 has also been identified as being a component of the insoluble organic matrix^10^, suggesting a close association between that and the silicalemma.

BLASTp results (Table S2 and Figs S3a, S4a) indicate that the TpSAPs are members of a family conserved among diatoms, predominantly amongst centric species although two SAPs are found in the araphidic pennate species *F*. *cylindrus*. TpSAP1 and TpSAP3 C-terminal GFP transformant lines displayed similar localization patterns concentrated on the sites of forming valves and girdle bands (Figs 3, 4, 5). Co-localization of TpSAP3-GFP and silica showed that TpSAP3 extends beyond the edge of the forming silica (Fig. 4g), consistent with an association with the SDV membrane. TpSAP3 undergoes proteolytic processing in a time-dependent manner, generating two cleavage fragments (Fig. 6). The amount of each band, relative to total protein, is maximal during valve formation at 6 h and then decreases, which corresponds to a similar pattern in the transcript data, with the protein response lagging 1 h behind (Fig. 2). The inverse relationship between the relative abundance of the 70 kDa and 37 kDa bands shows that the full length TpSAP3 protein is proteolytically cleaved during valve formation (Fig. 6d). The size of the 37 kDa band indicates this cleavage occurs in the cytosol after the TM domain (Fig. 6b). No known proteolytic cleavage sites were identified between the TM and the conserved domain rendering this cleavage mechanism unknown. The relative abundance of the doublet occurring at approximately 25 kDa remains fairly constant throughout the time course (Fig. 6d). The cleavage site for this fragment seems to correspond with the C-terminal RXL motif (Fig. 6b). Thus far, RXL cleavage sites have only been found in biosilica associated proteins (silaffins, silacidins, cingulins and frustulins)^7,11,12,51^. It has been hypothesized that RXL sites are involved in precursor peptide processing, though this remains to be proven^7,11,12^. N- terminal RXL sites are found in most biosilica associated proteins, except cingulins Y, and correspond to the cleavage site of the pro-peptide^52^.

Transmembrane proteins that undergo cleavage have also been implicated in coral skeleton biogenesis^53^. Ramos-Silva and colleagues identified multiple TM containing peptides in the *Acropora millepora* proteome. Skeleton biogenesis occurs extracellularly in corals and mass spectrometry determined that only the extracellular N-terminal portion of these proteins remained associated with the skeleton, suggesting that the TM and C-terminus are cleaved during skeleton biogenesis, subsequently leaving the N-terminus to be incorporated into the skeleton. Similarly, our evidence demonstrates that the intralumenal N-terminus of TpSAP3 remains embedded within the silica while the C-terminus is cleaved. In contrast to the model for *A*. *millepora* though, TpSAP3 cleavage does not release the TM from the embedded N-terminus.

The TpSAP3 C-terminal GFP constructs were also localized to spherical intracellular vesicles which were present concurrently with the SDV and forming silica structures (Figs 3, 4). The origin and role of this compartment is unclear. One possibility is that it is an intermediate compartment, delivering membrane and proteins for the growth of the SDV, although previous electron microscopy suggested that numerous smaller vesicles were involved^54^. A recent study has identified vesicles that traffic another silicalemma associated protein, Sin1, to the SDV^50^. The increase in fluorescence intensity of this compartment during silicon starvation could result from accumulation of proteins intended for cell wall morphogenesis (Fig. S6). The vesicle is also found in close association with the nucleus in dividing cells (Fig. S5). The proximity of the vesicle to the nucleus and associated membrane networks may facilitate its enrichment in secreted proteins. The visual absence of this compartment when GFP is inserted in the intralumenal part of TpSAP3 could be explained by the effects of pH. The SDV lumen is acidic^55^ and it has been suggested that GFP fluorescence is quenched in these conditions^56^. Silaffin-GFP fusion proteins have not been observed in an intracellular compartment, but subcellular fractionation and Western blot analysis using a GFP antibody confirm their presence in Golgi and Endoplasmic Reticulum containing fractions^56^. Our data support the concept that the fluorescently labeled compartment could serve as an intermediate to the SDV, but more detailed time course imaging is required to further evaluate. Despite other similarities in localization, TpSAP1 was not found localized to intracellular compartments other than the SDV, possibly indicating differences in its function or mode of transport to the SDV.

TpSAP2-GFP fluorescence was not observed associated with the silicalemma or any specific membrane system; rather it was localized to the cytosol (Fig. 3d). Since it is unlikely a predicted TM protein would be found outside a membrane, this suggests that the C-terminal region (containing GFP) may be rapidly cleaved from the protein and remain in the cytosol. TpSAP2 differs substantially in its gene expression pattern compared withTpSAP1 and 3 (Fig. 2). Comparison of expression patterns suggests that TpSAP2 is present in the cell prior to accumulation of TpSAP1 and 3. Also in contrast to TpSAP1 and 3, which have acidic isoelectric points (5.45 and 5.2), TpSAP2 has a PI of 11.3 (Table S2). More extensive characterization is required to clarify the localization and role of TpSAP2 but it seems to play a distinct role from TpSAP1 and 3.

**Figure 9:**
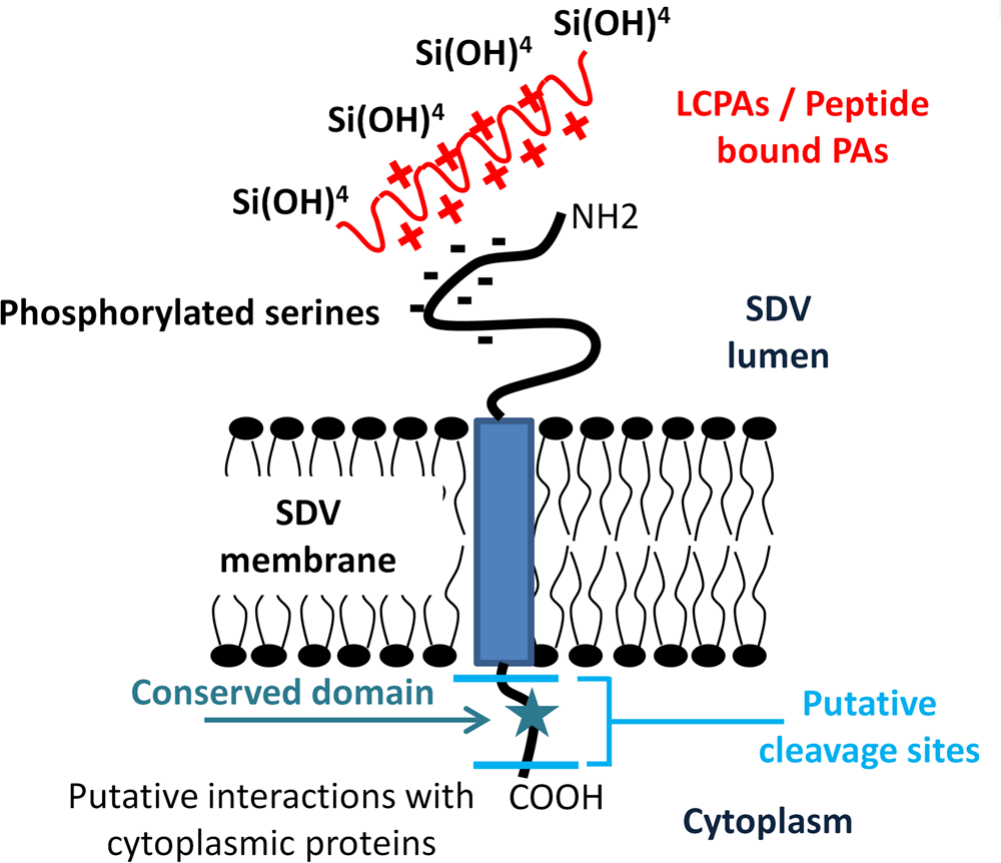
Schematic representation of SAP organization relative to the SDV membrane. Putative interactions of phosphorylated serines with Long Chain Polyamines (LCPAs) or peptide bound polyamines (PAs) and silicic acid are shown inside the SDV lumen. Putative cleavage sites of cleavage and interactions between cytoplasmic proteins and the conserved domain are shown in the cytoplasm.

Our results suggest the SAPs have a role in the process of cell wall morphogenesis. The TpSAPs do not contain the KXXK motifs present in silaffins and cingulins, which are associated with silica polymerization activity^57^; this suggests that TpSAPs may not be directly involved in the initiation of silicification. However the intralumenal portions of the TpSAPs are enriched in serine residues which Western blot band size (in TpSAP3) and posttranslational prediction tools suggest are likely phosphorylated. Previously characterized cell wall associated proteins like silaffins and cingulins are also enriched in serines which are phosphorylated^58^. *In vitro* experiments have shown that silaffin phosphorylation is important for the self-assembly of silaffins and polyamines in absence of an anionic buffer^59^. It is hypothesized that negatively charged phosphate groups mediate interactions with the positively charged peptide bound polyamines present in silaffins and long chain polyamines (LCPAs)^60^. By analogy, the proposed phosphate groups on the TpSAPs could play the same role facilitating silicification through charge interactions, rather than initiating it (Fig. 9).

The altered silica morphologies in TpSAP1 and TpSAP3 knockdown lines allow us to address their roles in silica structure formation. Although both proteins impact silicification on the valve distal surface, knockdown of each produces a distinct phenotype (Figs 8, S8, S9). From this we hypothesize that each performs a different function in silica structure development and we have therefore generated two conceptual models for what their roles in the cell may be, based on our current understanding of the components involved.

Knockdown of TpSAP3 transcripts resulted in little to no valve distal surface silica deposition, leaving valve base layer exposed (Figs 8, S9). In *T*. *pseudonana* the native distal surface patterning is characterized by an interconnected network of ridges positioned along the pre-defined ribs of the base layer (Fig. 8e,f). We hypothesize that TpSAP3 plays an essential role in the formation of the silica ridges through the aggregation of silica polymerizing elements (silaffins and LCPAs) along the base layer ribs. Previous work with LCPAs and silaffins^52^ suggest they form interconnected structures in a continuous LCPA/protein network which can build on itself as silicification proceeds. Charge interactions between phosphorylated serines in TpSAP3 and a LCPA/protein network in the base layer could enhance silica precipitation and contribute to the formation of ridges (Fig. 9).

Valves in TpSAP1 knock down lines are characterized by mislocated pattern centers (Fig. 8b) and aberrant patterning of the distal surface silica network (Fig. 8a,b). Although the imaging shows that valves with mislocated pattern centers frequently have more abnormal distal surfaces, a mislocated pattern center is not required for an altered surface network (Fig. S8). This implicates TpSAP1 in the control of two separate processes, distal surface silica deposition and pattern center positioning. A decrease in available TpSAP1 in knockdown lines may alter the availability of phosphorylated serines in the SDV lumen (Fig. 9), and this may affect the native network deposition process. Previous work has demonstrated that the location of the primary site of silicification (PSS) determines the pattern center location in both centric and pennate species^54^. The positioning of the PSS is likely influenced by the microtubule organizing center which has been localized there and is associated with its movement^14,54^. The influence of TpSAP1 over the positioning of the pattern forming center, suggests that it may interact directly or indirectly with a proximal surface microtubule network.

A potential role for the cytoplasmic conserved domain found in all TpSAPs could be to facilitate interactions between the SAPs and cytoplasmic proteins (Fig. 9). Cytoskeletal proteins have been shown to play a significant role in diatom cell wall morphogenesis^14,54^. A previous model proposed interaction of silicalemma-associated proteins with the cytoskeleton to explain controlled silica deposition^19^. It is possible that the SAP conserved domain could mediate such interactions. Though the conserved domain does not match any known cytoskeleton interacting domains, a recent paper has shown that diatom actin related and actin binding proteins do not always adhere to the canonical sets, this could explain a novel binding domain^61^. Another possibility is that the conserved domain does not interact directly with cytoskeletal proteins, but with other cytosolic proteins, these multi-protein complexes may then interact with the cytoskeleton.

The ability of diatoms to control the deposition of silica with high precision and reproducibility in a membranous compartment is unique. Deciphering the genetic basis of how diatoms make reproducible structures is an important step to elucidate this process. Several soluble proteins able to precipitate silica have been discovered over the last few decades. However, the mechanisms by which the final three-dimensional cell wall patterns are formed remain unknown. This work describes a family of transmembrane proteins localized to the SDV membrane and involved in mesoscale silica structure formation and patterning, as well as the first genetic alterations of silica structure through the manipulation of individual genes. The demonstration of specific phenotypes generated with knockdowns of TpSAP1 and 3 opens the door towards characterizing their roles in more detail.

Silica cell wall formation is a complex cellular process, involving hundreds of genes and their encoded proteins, whose efficacy may often rely on interactions with one another. Genetic-based approaches will be essential to unravel this process. Although we do not have the ability to do classical genetic crosses on diatoms, we can knockdown, knock out, and over express individual genes. The data presented herein, demonstrates that knocking down a single gene is sufficient to generate a consistent phenotypic change in silica structure, which in turn can facilitate our understanding of a specific a protein’s role. This sets the stage for examining novel domains and other candidate proteins involved in the process of diatom cell wall formation.

## Electronic supplementary material

Supplemental Information
