# Supplementary material for: Characterization of a New Protein Family Associated With the Silica Deposition Vesicle Membrane Enables Genetic Manipulation of Diatom Silica
Source: Sci Rep. 2017 Oct 18;7:13457. doi: 10.1038/s41598-017-13613-8 (PMC5647440; doi:10.1038/s41598-017-13613-8)
Supplement: Supplementary file 1 — Supplemental Information [file 41598_2017_13613_MOESM1_ESM.pdf]

Supplementary Information

Characterization of a New Protein Family Associated With the Silica Deposition Vesicle Membrane Enables Genetic Manipulation of Diatom Silica

Benoit Tesson[1,*], Sarah J.L. Lerch[1] and Mark Hildebrand [*]

Marine Biology Research Division, Scripps Institution of Oceanography, University of California San Diego, La Jolla, California, United States of America.

[1] These authors contributed equally to this work.

Author contact information:
*B. Tesson: tessonben@gmail.com
S. Lerch: slerch@ucsd.edu
*M. Hildebrand: mhildebrand@ucsd.edu

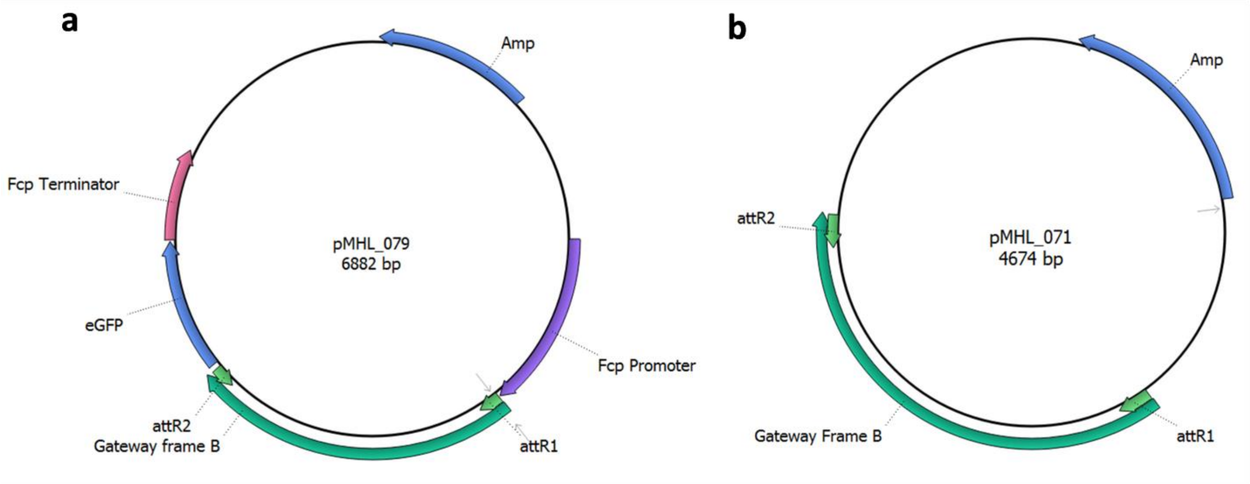

Figure S1: Plasmid maps showing destination vectors used for (a) fcp expression and (b) native expression constructs. Gateway frame B and att sites indicate insertion site for target sequence. (Amp: region encoding ampicillin resistance).

TPSAP3_Intralumenal GFP

MMQRFALLVLLALSANAEQASLRSSRQLYNSNSSSSSSSSSSSGWSFWGSLLQLLDHVHAPCPPGPLHHKDENGDPLKRGECWSDLMVSKGEEL
FTGVVPILVELDGDVNGHKFSVSGEGEGDATYGKLTLKFICTTGKLPVPWPTLVTTLTYGVQCFSRYPDHMKQHDFFKSAMPEGYVQERTIFFKDD
GNYKTRAEVKFEGDTLVNRIELKGIDFKEDGNILGHKLEYNYNSHNVYIMADKQKNGIKVNFKIRHNIEDGSVQLADHYQQNTPIGDGPVLLPDNH
YLSTQSALSKDPNEKRDHMVLLEFVTAAGITLGMDELYKHHPPKPKKKSSSSHSSSSKSKVTYSACEQGEDGCFENVNCADGGDCNEYIKCVDQD
GNAVDCFTNDDGQNIGGNGWKDDGYSVTDDATDDNSATDDAQNVVDDQWGSDGWSASNLDSGNSFASKSGNKSTPVWPFIVGALVAGVI
GAAFIVSRRKRREEDSHPLDGSIKKRQKLFSGFSRNKKGALNEDFDNEEGKPNFIEIGEHNKRTNSYAAPRNLVDDNSEYSR*

Figure S2: TpSAP3 amino acid sequence with GFP (underlined) inserted in the intraluminal region. Signal peptide is in dark teal, RXL domains in orange, serine rich regions in blue, transmembrane domain in red.

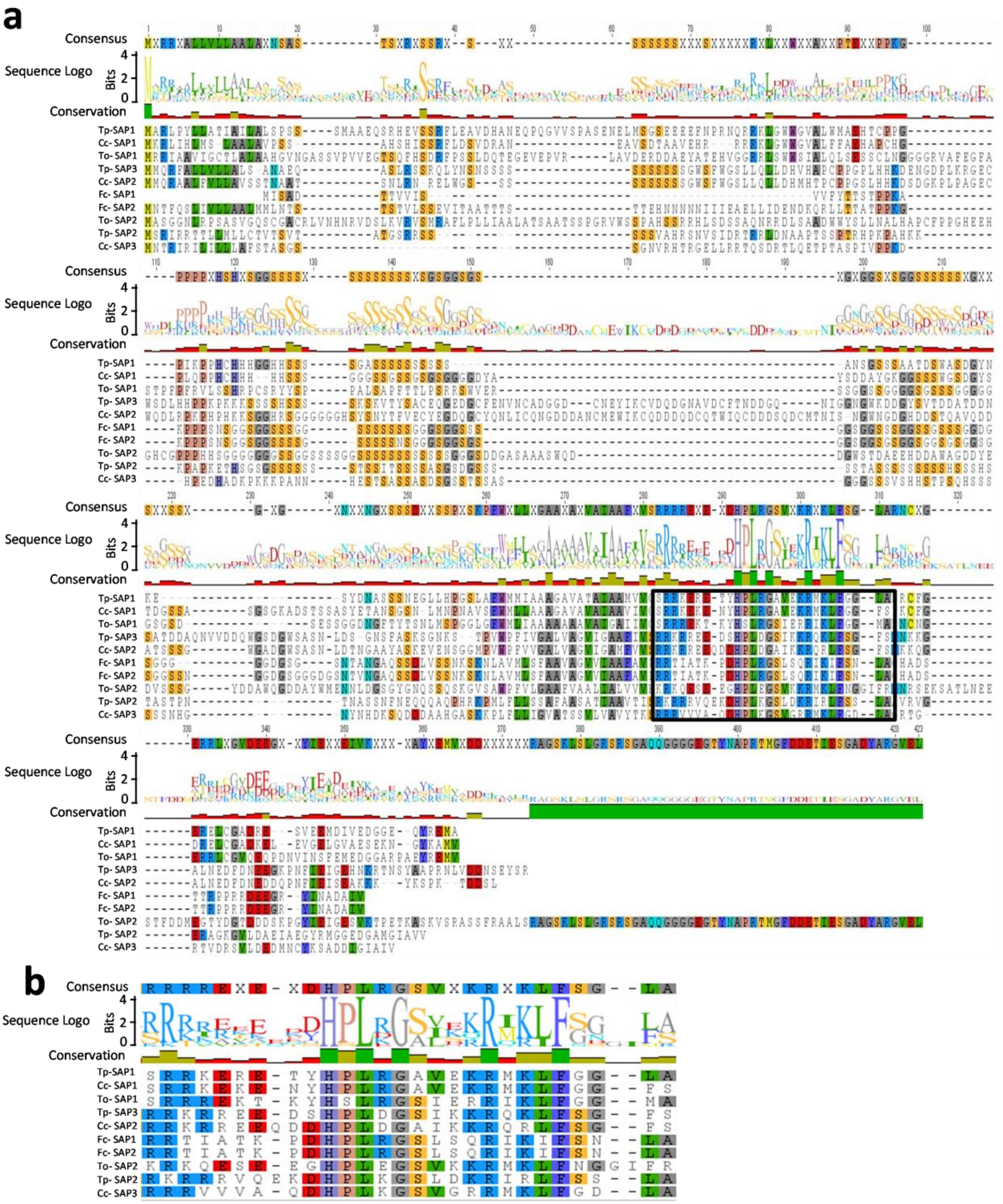

Figure S3: (a) Alignment of SAP protein sequences from *T. pseudonana* (Tp), *T. oceanica* (To), *F. cylindrus* (Fc), and *C. cryptica* (Cc). Conserved domain is boxed in black. (b) Alignment of conserved domains. Amino acids shared with the consensus sequence are highlighted in parts a and b.

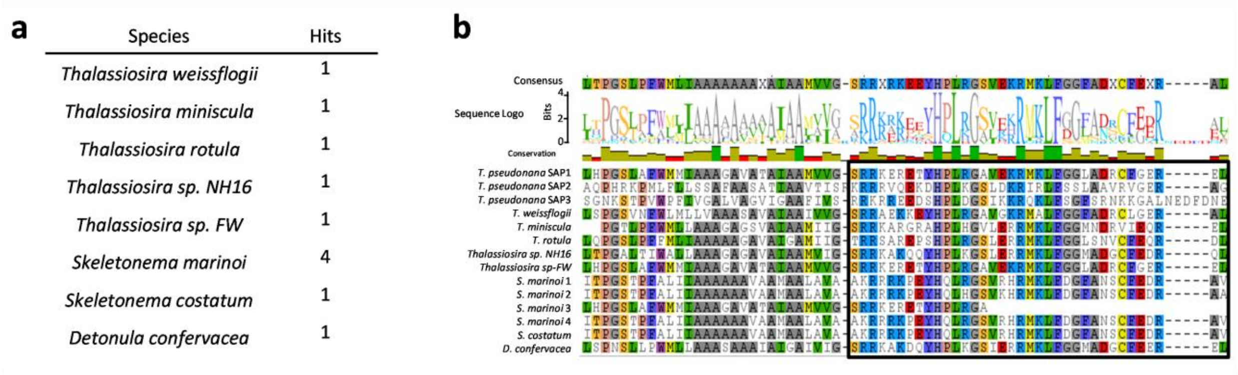

Figure S4: a) Table displaying distinctive hits to TpSAP1 from MMETSP database. b) Alignment of sequences from TpSAPs and MMETSP database hits over a portion of the TM domain and the conserved domain (outlined in black). Amino acids shared with the consensus sequence are highlighted. Redundant hits from different strains of the same species have been removed if 100% sequence alignment was present.

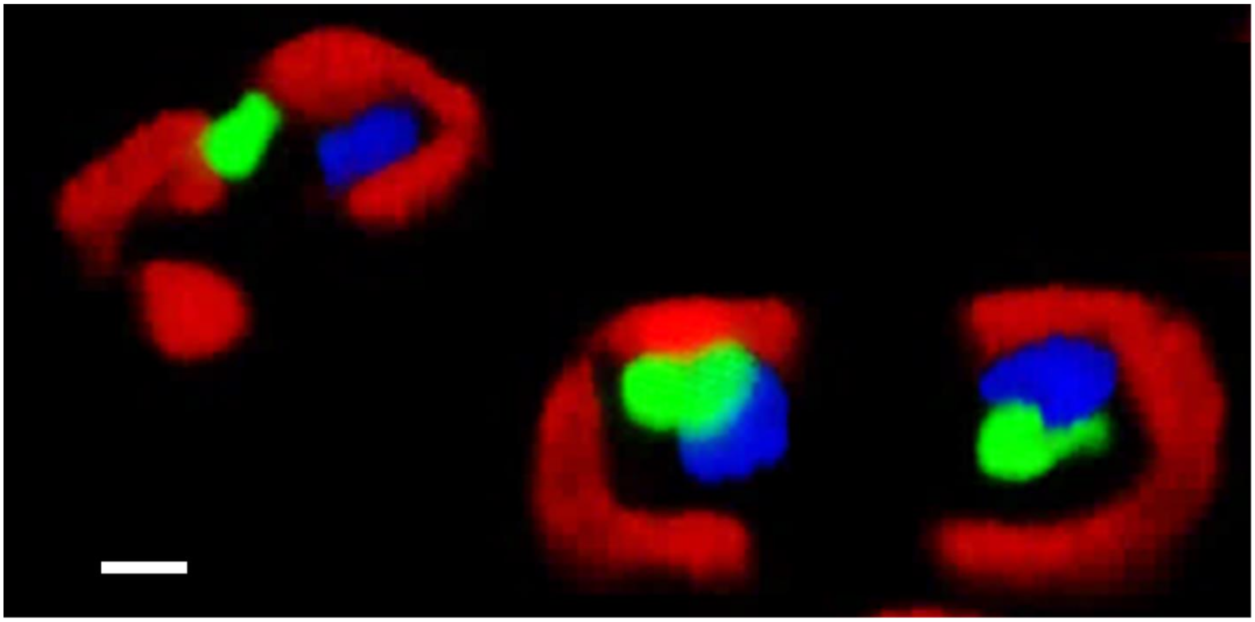

Figure S5: 3D reconstruction of a cell expressing SAP3-GFP under control of the native promoter stained with DAPI. GFP is green, DNA is blue, and chlorophyll autofluorescence is red. Scale bar is 2 µm.

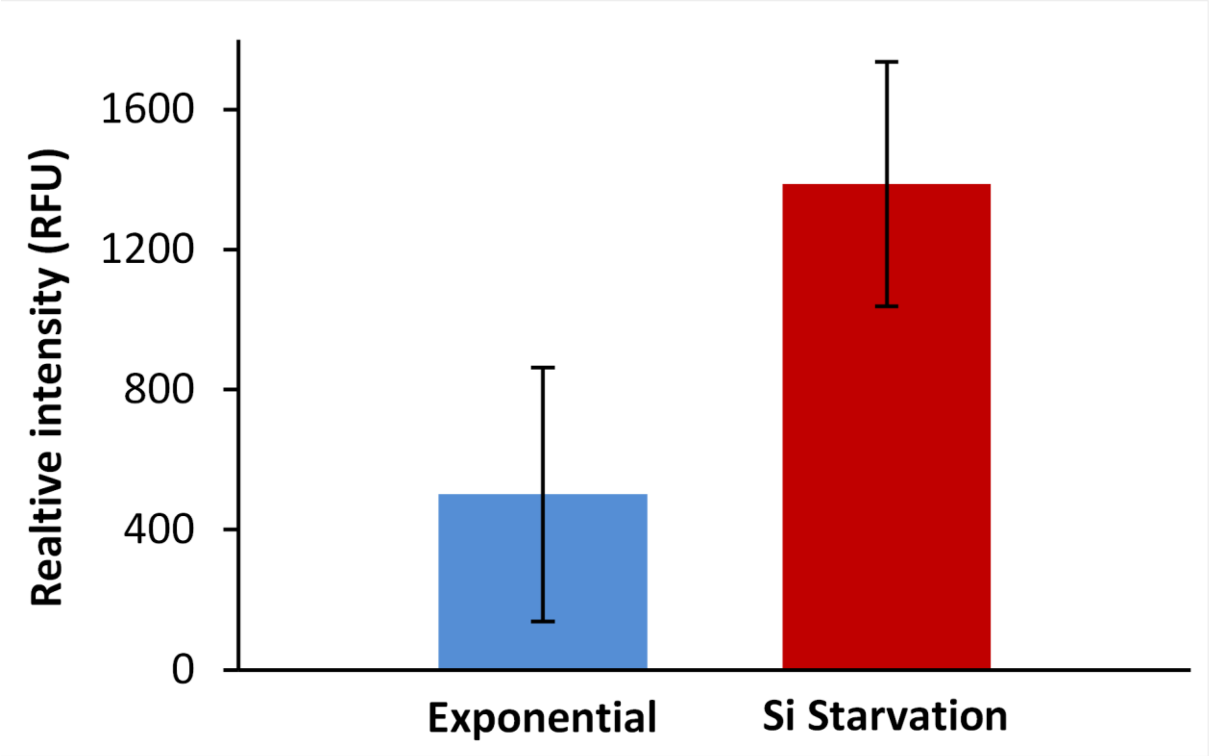

Figure S6: Average relative GFP fluorescence intensity of fluorescent intracellular compartment in cells expressing TpSAP3-GFP fusion protein. Intensity during exponential growth (blue) and after 4 h silicon starvation (red). Error bars represent standard deviation. $p<0.01$; $n=24$.

TpSAP1

MARLPYLLATIAILALSPSSSMAAEQSRHEVSSRFLEAVDHANEQPQGVVSPASENELMSGSEEEEFNPRNQRRKLGWWGVALWMAEHTCPP
GPIKPPHCHHHGGHHSSSSGASSSSSSSSSSANSGSSSAATDSWASDGYNKESYDNASSSNEGLLHPGSLAFWMMIAAAGAVATAIAAMVVGS
RRKERETYHPLRGAVEKRMKLFGGLADRCFGERELCGADRESVEEMDIVEDGGEQYREMA*

TpSAP2

MSRIRRTTLLMLLCTVTSVTATGSRRSSSSSVAHRSNVSIDRTRRLDNAAPTSSPTRHPKPAHKKKPAPKETHSGSGSSSSSSSTSSITSSSSASGSD
GSSSSSTASSSSSSSSSHSSSHSTASTPNTNASSNFNEQQQAQPHRKPMLFLLSSAFAASATIAAVTISRKRRRVQEKDHPLKGSLDKRIRLFSSLAA
VRVGERAGKGVLDAEIAEGYRMGGEDGAMGIAVV*

TpSAP3

MMQRFALLVLLALSANAEQASLRSSRQLYNSNSSSSSSSSSSSGWSFWGSLLQLLDHVHAPCPPGPLHHKDENGDPLKRGECWSDLHHPPKPKK
KSSSSHSSSSKSKVTYSACEQGEDGCFENVNCADGGDCNEYIKCVDQDGNAVDCFTNDDGQNIGGNGWKDDGYSVTDDATDDNSATDDAQN
VVDDQWGSDGWSASNLDSGNSFASKSGNKSTPVWPFIVGALVAGVIGAAFIVSRRKRREEDSHPLDGSIKKRQKLFSGFSRNKKGALNEDFDNE
EGKPNFIEIGEHNKRTNSYAAPRNLVDDNSEYSR*

Figure S7: TpSAP sequences highlighting predicted glycosylation and phosphorylation sites. Blue: glycosylation sites, underlined: phosphorylation sites.

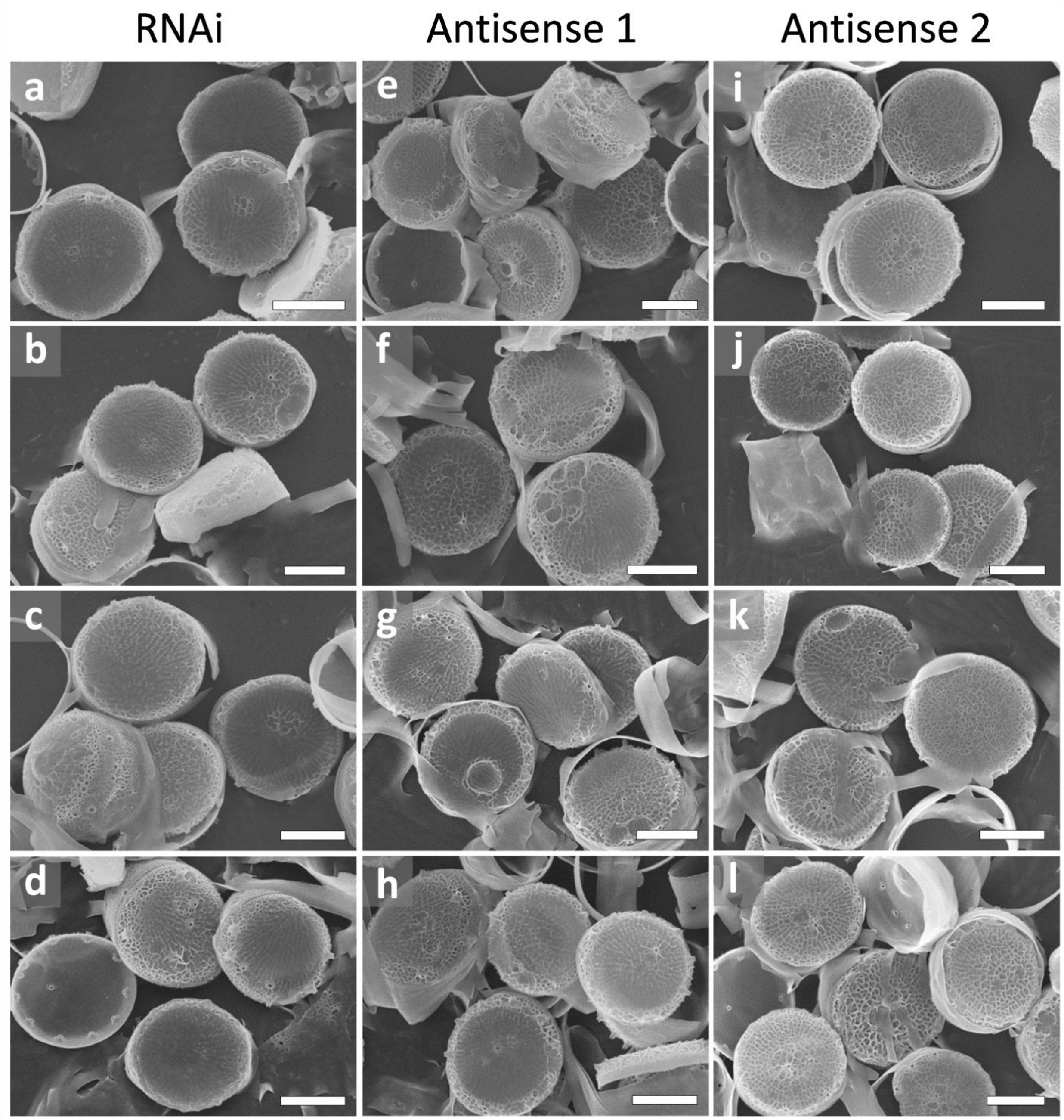

Figure S8: Scanning electron micrographs of valves synthesized by a subset of evaluated TpSAP1 RNAi (a-d) and antisense knockdown lines (e-l). Numbers indicate different clonal lines. Scale bars are 2μm.

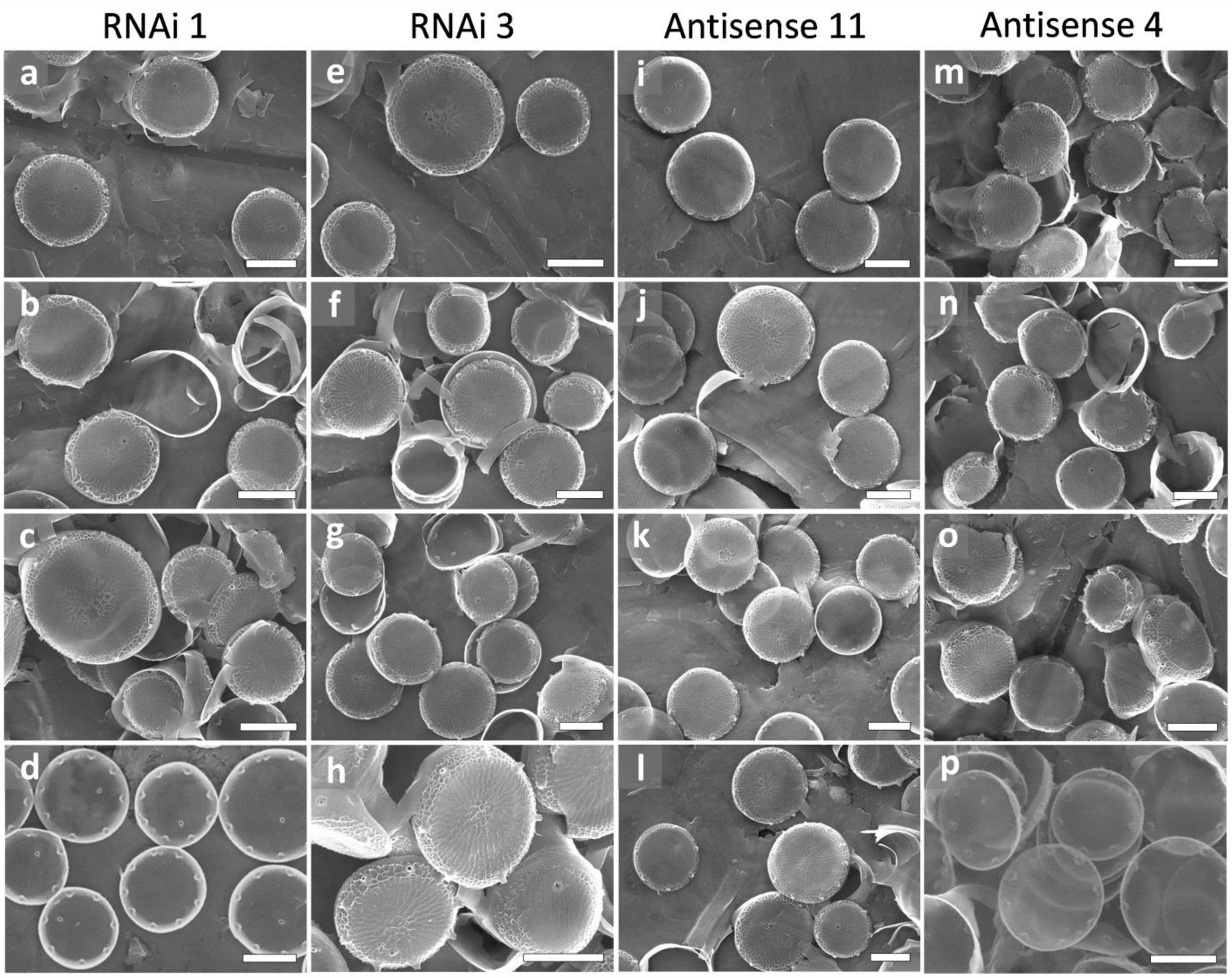

Figure S9: Scanning electron micrographs of valves synthesized by a subset of evaluated TpSAP3 RNAi (a-h) and antisense (i-p) knockdown lines. Numbers indicate different clonal lines. Proximal valve surface views shown in d and p. Scale bars are 2µm.

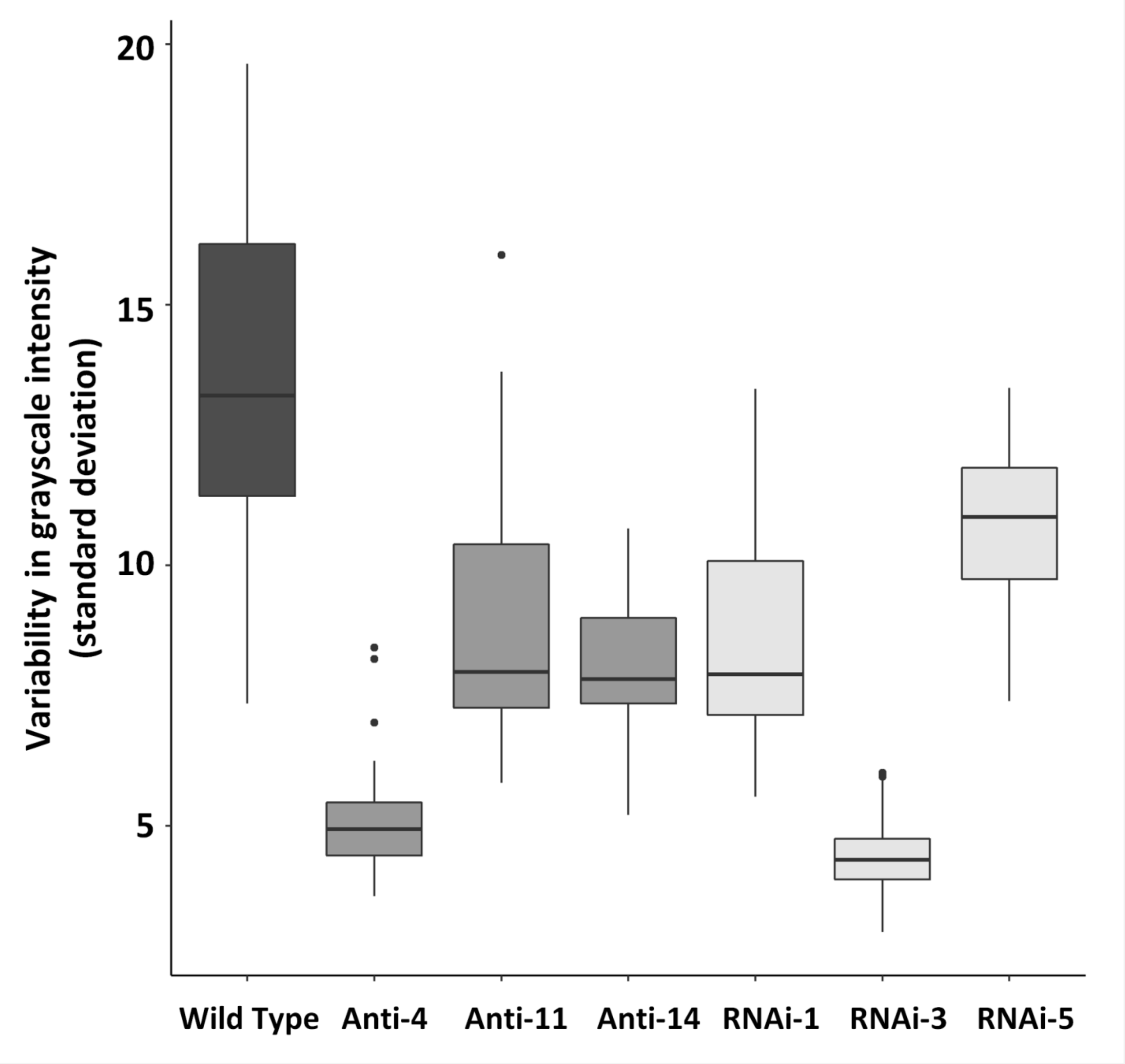

Figure S10: Box plot displaying average standard deviation of valve surface gray scale intensity in wild type and TpSAP3 antisense (Anti) and RNAi knockdown lines. Numbers indicate different clonal lines. All knockdown lines except for RNAi-15 were significantly different from wild type as determined by Games-Howell post hoc analysis. Adjusted p values < 0.002; n=20.

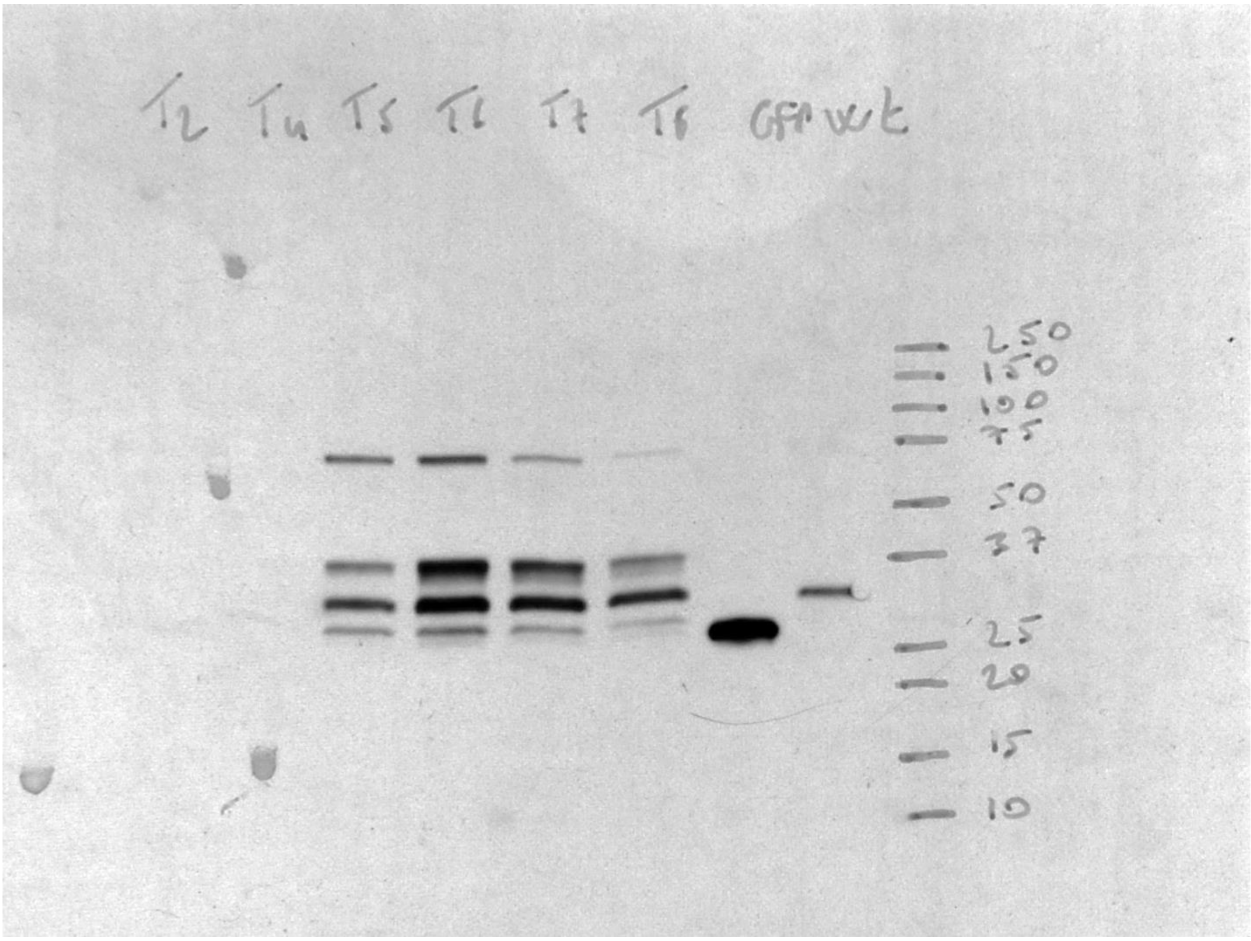

Fig. S11. Unprocessed image of Western blot used in Fig. 6

Table S1. Primers used to amplify target genes for cloning, att sites not included.

Description	Name	25736	25805	25807	GFP
Gene	**FcpF**	ATGGCACGCCTTCCCTACC	ATGTCGAGAATAAGAAGAACAACA	ATGATGCAACGATTCGCCCTC	ATGGTGAGCAAGGGCGAGGA
Gene	**FcpR**	AGCCATCTCCCTATACTGCTC	CACCACAGCAATCCCCATGG	ACGGCTGTATTCACTGTTATCA	TTACTTGTACAGCTCGTCCATGC
Native promoter+gene	**Nat1-F**	CTGCTGCTTCTGCGCTACGTA	TCTTGTGTTTTGATGAAATTGGTAA	GAAAGATTTGTAGTGATGTCAAAG	
Native promoter+gene	**Nat1-R**	AGCCATCTCCCTATACTGCTC	CACCACAGCAATCCCCATGG	ACGGCTGTATTCACTGTTATCA	
Native terminator	**Nat2-F**	AGGGTTGATGTGTATGGTGTA	AGAATGATACAGTGTATGATATGAA	GAAGCTGTGTTGTTCGAGTCG	
Native terminator	**Nat2-R**	TTACATGTCGACGAAAGGATG	CTTTCAATCCGAGTACGGGCA	AGCATCCACAAGGCCAAAGG	
Gene with GFP in the intraluminal portion	**GFPIn1-F**			ATGATGCAACGATTCGCCCTC	ATGGTGAGCAAGGGCGAGGA
Gene with GFP in the intraluminal portion	**GFPIn1-R**			CAAATCAGACCAGCACTCTCC	CTTGTACAGCTCGTCCATGC
Gene with GFP in the intraluminal portion	**GFPIn2-F**			CACCATCCTCCTAAACCCAAG	
Gene with GFP in the intraluminal portion	**GFPIn2-R**			TTAACGGCTGTATTCACTGTTATC	
Knockdown RNAi, sense portion	**RNAi1F**	TCACTCTCCGCCGACTTTCC		ATGATGCAACGATTCGCCCTC	
Knockdown RNAi, sense portion	**RNAi1R**	CTGTCATTCCATCGTTGGTTG		CTGTAGTGTTTTGAGTTGGAGA	
Knockdown RNAi, antisense portion	**RNAi2F**	CCATCCAAAGAGCGACTCC		CCTGCTGACAATAAAGGCGG	
Knockdown RNAi, antisense portion	**RNAi2R**	TCACTCTCCGCCGACTTTCC		ATGATGCAACGATTCGCCCTC	
Knockdown antisense	**AntiF**	CGATGATGACGACGACGACG		TACCATACATGACGTGTCATCTTA	
Knockdown antisense	**AntiR**	TCACTCTCCGCCGACTTTCC		CGCAAGCGTCGTGAAGAAGAC	

Fcp, target under fcp promoter. Nat, target including native promoter (Nat1) and terminator (Nat2) sequences. GFPIn, C term (GFPIn1) and N term (GFPIn2) portions of target for internal GFP construct. Fcp: fucoxanthin chlorophyll a/c-binding protein gene promoter, GFP: Green fluorescent protein.

Table S2: Characteristics of SAP proteins identified from *T. pseudonana*, *F. cylindrus, T. oceanica* and *C. cryptica* genome databases using BLASTp.

	**Protein ID**	**MW**	**PI**	**% Serine**	**TM**	**P**	**G**	**RXL**
**TpSAP1**	25736	26.31	5.45	15.5	1	42	30	3
**TpSAP2**	25805	23.92	11.33	24.8	1	65	58	1
**TpSAP3**	25807	34	5.2	15	1	51	37	2
**FcylSAP1**	249181	17.13	9.77	23.5	1			0
**FcylSAP2**	277621	22.9	6.83	18.8	1			1
**TocSAP1**	10337	29.15	6.24	13.7	1			1
**TocSAP2**	31902	40.02	5.32	17.2	1			2
**CcSAP1**	g4294	25.2	6.89	15.3	1			2
**CcSAP2**	g9622	33.9	4.9	11.9	1			1
**CcSAP3**	g8103	22.6	9.5	17.4	0			1

Tp: *T. pseudonana*, Fcyl: *F. cylindrus*, Toc: *T. oceanica*, Cc*: C. cryptica*, MW: Molecular weight, PI: Isoelectric point, TM: Transmembrane domain, P: phosphorylation sites, G: glycosylation sites.
